# Expression of the *Helicobacter pylori* adhesin SabA is controlled via phase variation and the ArsRS signal transduction system

**DOI:** 10.1099/mic.0.2007/016055-0

**Published:** 2008-08

**Authors:** Andrew C. Goodwin, Daniel M. Weinberger, Christopher B. Ford, Jessica C. Nelson, Jonathan D. Snider, Joshua D. Hall, Catharine I. Paules, Richard M. Peek, Mark H. Forsyth

**Affiliations:** 1 Department of Biology, The College of William and Mary, Williamsburg, VA 23187-8795, USA; 2 Division of Gastroenterology and Department of Cancer Biology, Vanderbilt University School of Medicine, Nashville, TN 37232-2279, USA; 3 Department of Veterans Affairs Medical Center, Nashville, TN 37212, USA

## Abstract

Adaptation to the acidic microenvironment, and adherence to mucosal epithelium, are essential for persistent colonization of the human stomach by *Helicobacter pylori*. The expression of SabA, an adhesin implicated in the ability of *H. pylori* to adhere to the host gastric epithelium, can be modulated by phase variation via slipped-strand mispairing in repetitive nucleotide tracts located in both the promoter region and the coding region. This study demonstrates the occurrence of phase variation at the *sabA* locus within individual strains of *H. pylori*, and among multiple isolates from a single patient. In addition, transcription of *sabA* is repressed by the acid-responsive ArsRS two-component signal transduction system *in vitro*. Our results demonstrate that isogenic inactivation of the *arsS* (*jhp0151*/*HP0165*) histidine kinase locus results in a 10-fold SabA-dependent increase in adherence to gastric epithelial cells in strain J99 (contains an in-frame *sabA* allele), but not in strain 26695 (out-of-frame *sabA* allele). The combination of transcriptional regulation of the *sabA* locus by the ArsRS two-component signal-transduction system and the generation of subpopulations harbouring alternate *sabA* alleles by slipped-strand mispairing during chromosomal replication could permit *H. pylori* to rapidly adapt to varying microenvironments or host immune responses. As a pathogen with a paucity of regulatory proteins, this dual regulation indicates that SabA expression is a tightly regulated process in *H. pylori* infection.

## INTRODUCTION

*Helicobacter pylori* is a Gram-negative bacterium that infects more than half the world's population, and it colonizes the human gastric epithelium. Colonization generally occurs in childhood and, without treatment, persists for the lifetime of the host. As one of the most genetically diverse bacterial species, *H. pylori* has co-evolved with human hosts, and generated populations that productively colonize a particular gastric niche. Although many colonized individuals remain asymptomatic, *H. pylori* is a major aetiological agent of peptic ulcer disease, and a recognized risk factor for gastric cancer (Blaser & Berg, 2001; Merrell & Falkow, 2004; Kusters *et al.*, 2006).

Adherence to host cell receptors protects *H. pylori* from clearance during mucus shedding, and ensures consistent access to nutrients released by damaged gastric epithelial cells, facilitating long-term colonization, and potentially contributing to disease onset (Gerhard *et al.*, 1999; Odenbreit, 2005; Aspholm *et al.*, 2006). *H. pylori* has several well-characterized adhesins, including BabA, which binds to the Lewis B (Le^b^) antigen (Boren *et al.*, 1993; Ilver *et al.*, 1998), and SabA, which binds to glycosphingolipids displaying a sialyl-dimeric Lewis X (sialyl-Le^x^) antigen (Mahdavi *et al.*, 2002).

Glycoconjugates bearing the sialyl-Le^x^ antigen are rarely expressed in healthy gastric epithelial cells (Madrid *et al.*, 1990), but they are upregulated during inflammation, and serve as binding sites for host cell adhesins of the selectin family (Alper, 2001). Accordingly, SabA-mediated adherence is positively correlated with sialyl-Le^x^ concentration *in vitro* (Linden *et al.*, 2004), and colonization density is increased in patients who produce high levels of sialyl-Le^x^, or are infected with SabA-positive strains of *H. pylori* (Sheu *et al.*, 2006). *H. pylori* shows a tropism for areas of reduced acidity in the stomach that contain gastric pit cells producing sialyl-Le^x^ (Bjorkholm & Salama, 2003), and studies have shown that clearance of infection reduces production of sialyl-Le^x^ receptors to pre-infection levels (Mahdavi *et al.*, 2002; Acheson & Luccioli, 2004; Roche *et al.*, 2004).

Expression of bacterial adhesins can be regulated both by reacting to changes in the environment using signal transduction, and by generating genetic changes that affect production of functional proteins. It has been demonstrated that some *H. pylori* genes are regulated by phase variation (Saunders *et al.*, 1998; Salaun *et al.*, 2004), a mechanism by which genes can be expressed in an all-or-nothing manner. Phase variation can control gene expression at the transcriptional and the translational levels, and in other organisms it has been shown to mediate evasion of the host immune response, or to modify virulence properties (van der Woude & Baumler, 2004). At the level of chromosomal replication, a molecular mechanism of phase variation known as slipped-strand mispairing can insert or delete nucleotides within repetitive DNA tracts, usually near the 5′ end of genes. This results in altered reading frames, alternatively yielding truncated or full-length proteins (de Vries *et al.*, 2002).

The *sabA* locus contains a homopolymeric thymine (poly-T) tract in the promoter region, and a dinucleotide cytosine–thymine repeat (CT repeat) in the coding region. Studies have demonstrated that collections of *H. pylori* strains exhibit significant diversity in the presence of *sabA*, CT-repeat tract lengths, and resulting expression of SabA (Lehours *et al.*, 2004; Sheu *et al.*, 2006; Yamaoka *et al.*, 2006). This regulatory mechanism may explain why 1 % of J99 colonies in one study spontaneously lost their ability to bind sialyl-Le^x^ (Mahdavi *et al.*, 2002). Yamaoka *et al.* (2002) demonstrated that adherence, colonization ability, bacterial density, and induction of inflammation were all decreased when *sabA* or *sabB* was switched off, indicating that this mechanism of *sabA* regulation has functional significance.

Aside from genetic changes, *H. pylori* also uses two-component signal transduction (TCST) systems to respond to environmental changes. Activation of a TCST system results in changes in the rates of histidine kinase and response regulator protein phosphorylation, leading to altered promoter-region DNA-binding activity of the response regulator, and either positive or negative regulation of gene transcription (Beier & Frank, 2000; Stock *et al.*, 2000). A previous study in our laboratory utilized DNA macroarrays to define the set of genes regulated by the HP0165–HP0166 TCST system in *Helicobacter pylori* strain 26695 (Forsyth *et al.*, 2002). That study, which compared genome-wide transcriptional profiles between wild-type *H. pylori* and an isogenic HP0165 histidine kinase mutant, found one gene to be repressed in the null mutant, while six genes, including *sabA* (*HP0725*), were derepressed in the mutant.

Additional studies have further characterized and expanded this regulon, and identified acidic pH as the key environmental signal for *HP0165–HP0166*, and this locus has thus been redesignated *arsRS* (acid-responsive signalling) (Dietz *et al.*, 2002; Pflock *et al.*, 2004; Sachs *et al.*, 2006). *arsRS* has accordingly been demonstrated to be essential for the production of urease under acidic conditions (Panthel *et al.*, 2003), and is required for virulence in a mouse model (Pflock *et al.*, 2005). Transcription of the response regulator locus *arsR* (*HP0166*), an essential gene in *H. pylori* (Beier & Frank, 2000), is downregulated at pH 5.0 (Bury-Mone *et al.*, 2004) and non-phosphorylated ArsR has additional, phosphorylation-independent, regulatory activity (Schar *et al.*, 2005).

Recent studies have provided further insights into the relationship between pH and the expression of the genes regulated by ArsRS at the transcriptional and translational levels. Global gene expression analyses by Merrell *et al.* (2003) and Bury-Mone *et al.* (2004) have found that at pH 5.0 *sabA* and its paralogue *sabB* (de Jonge *et al.*, 2004) are downregulated, while HP1188, a novel *H. pylori* adhesin (Rubinsztein-Dunlop *et al.*, 2005), is induced. It has also been reported that SabA-positive status is associated with decreased acid secretion in patients, and that SabA protein levels are reduced at pH 5.0 (Yamaoka *et al.*, 2006).

In the present study, we hypothesized that the role of SabA in *H. pylori* adherence to AGS gastric epithelial cells is governed by phase variation and transcriptional regulation of *sabA* via the ArsRS system. We demonstrate that derepression of *sabA* transcription in an ArsS isogenic knockout strain of *H. pylori* (Forsyth *et al.*, 2002) results in a corresponding functional change in the ability of the bacterium to adhere to gastric epithelial cells. In addition, we demonstrate the existence of multiple alleles of *sabA* within a single *H. pylori* strain population, and among multiple isolates from a single patient, differing in the nucleotide-repeat tract lengths. Our findings provide new insights into the complex mechanisms regulating the expression of the SabA adhesin and may contribute to an improved understanding of persistent *H. pylori* infection, and thus have implications for development of therapeutics.

## METHODS

### Bacterial culture.

The *H. pylori* strains used in this study are described in Table 1. *H. pylori* was cultured on Trypticase Soy Agar II plates with 5 % sheep blood (BBL) at 37 °C and 5 % CO_2_, or Brucella agar plates supplemented with 10 % newborn calf serum (Gibco/Invitrogen). *Escherichia coli* DH5*α* was cultured in Luria–Bertani medium. When appropriate, media were supplemented with 100 μg ampicillin ml^−1^, 20 μg kanamycin ml^−1^, 25 μg chloramphenicol ml^−1^ (for *E. coli*), or 5 μg chloramphenicol ml^−1^ (for *H. pylori*).

### Amplified fragment length polymorphism (AFLP) analysis.

Oligonucleotides were designed to amplify the regions containing the poly-T (sabAampF and sabArev; see Table 2 for oligonucleotide sequences) and CT-repeat (sabAForC and HP0725R) tracts, as well as a downstream control region (sabABCFw and sabABCon). A 6-carboxyfluorescein (FAM) label was added to the 5′ end of one primer from each oligonucleotide pair. PCRs for AFLP analysis were performed using Vent High-Fidelity DNA polymerase (New England Biolabs). Amplicons were purified (QiaQuick PCR purification kit; Qiagen), processed at the DNA Sequencing and Synthesis Facility (Iowa State University, Ames, IA, USA), and analysed using genescan software (Applied Biosystems).

### Sequencing of *sabA* clones.

The *sabA* poly-T region was amplified by PCR from *H. pylori* strains 26695 and J99 with primers sabAampF and sabArev. A population of clones (denoted as pSabA-T) was generated by cloning the resulting PCR products in pGEM-T Easy. Similarly, the *sabA* CT-repeat tract region was amplified from *H. pylori* 26695 genomic DNA (gDNA) or cDNA with primers HP0725ForwII and HP0725Ext, and cloned to create a collection of clones denoted as pSabA-gDNA-CT and pSabA-cDNA-CT, respectively. Plasmid DNA was isolated using the QiaSpin Miniprep kit (Qiagen) or Wizard Plus Midiprep kit (Promega), and clones were sequenced as described below to determine the length of the poly-T or CT-repeat tracts.

### DNA sequencing.

Sequencing reactions were performed using the Big Dye v3.1 system (Applied Biosystems), purified over DTR gel-filtration spin columns (Edge Biosystems), vacuum-dried, and resuspended in Hi-Di Formamide (Applied Biosystems). Denatured samples were sequenced on an ABI 3100 Avant (Applied Biosystems), and analysed with Sequencing Analysis 5.1.1 (Applied Biosystems) and MacVector 7.0 (MacVector) software.

### Quantitative real-time PCR.

RNA was extracted from exponential-phase *H. pylori* cultures using the Invitrogen RNA Extraction System, treated with Turbo DNase (Ambion), and assayed for gDNA contamination by PCR. cDNA was synthesized from 2 μg RNA using random hexamers (Applied Biosystems) and AMV reverse transcriptase (Promega), purified using the QIAquick Nucleotide Removal kit (Qiagen), and verified via PCR. Quantitative real-time PCR was performed using the iCycler iQ real-time PCR detection system and SYBR Green supermix reagents (Bio-Rad). Relative expression of *sabA* (primers HP0725fwd and HP0725rev), *HP0218* (control gene; primers HP0218forw and HP0218rev) and *gyrB* (normalization gene; primers gyrB forw and gyrB rev) was calculated for J99 and the J99-*arsS* : : *cat* mutant strain. PCRs were performed in triplicate, and melt-curve analysis was used to ensure that a single product was amplified with each primer set. Differences in gene expression from three independent experiments were calculated by the ΔΔ*C*_T_ method (Livak & Schmittgen, 2001), and evaluated by Student's *t-*test.

### Creation of *H. pylori sabA* : : *cat*, *arsS* : : *cat*, *arsS* : : *km* and *arsS* complemented mutants.

A 525 bp fragment was amplified by PCR from the 5′ region of *H. pylori* J99 *sabA* using primers JCN725F and JCN725R, and cloned into pGEM-T Easy (Promega) to generate pJCN1. The chloramphenicol acetyltransferase gene (*cat*) was excised from pCM7 (a kind gift of Dr John Loh and Dr Timothy Cover, Vanderbilt University Medical Center, Nashville, TN, USA) and cloned into the pJCN1 *sabA Hin*dIII site, resulting in pJCN2. *H. pylori* strain J99 was naturally transformed with pJCN2, as previously described (Forsyth *et al.*, 2002), yielding strain J99-*sabA* : : *cat*. The *cat* cassette was also inserted into the *arsS Bgl*II site to generate strain J99-*arsS* : : *cat*.

*H. pylori* strains J99-165Km, J99-165Km-WT and J99-165Km-vector were a kind gift of Dr John Loh and Dr Timothy Cover (Loh & Cover, 2006). The *arsS* : : *km* mutant allele from J99-165Km was naturally transformed into passage-level-matched strains J99 and J99-*sabA* : : *cat* to generate strains J99-*arsS* : : *km* and J99-*arsS* : : *km*-*sabA* : : *cat* used in this study. Strain J99-*arsS* : : *km*-*rdxA* : : *arsS*, containing a complemented *arsS* allele, was constructed by natural transformation of the *rdxA* : : *arsS* allele from J99-165Km-WT into J99-*arsS* : : *km*, and selection on medium containing 15 μg metronidazole ml^−1^. The *arsS* : : *cat* mutant in a strain 26695 background has been described previously (Forsyth *et al.*, 2002).

### *H. pylori* adhesion to AGS cells.

The AGS (human gastric epithelial cell adenocarcinoma; ATCC) cell line was cultured in F12 Kaighn's medium supplemented with 10 % fetal bovine serum (Gibco/Invitrogen) at 37 °C and 5 % CO_2_. The cells were then used to seed 24-well plates with 2×10^5^ cells per well, and the plates were maintained at 37 °C and 5 % CO_2_ overnight. Plate-grown *H. pylori* was resuspended in 3.5 ml F12/10 % FBS, concentration was estimated by OD_600_, and the suspension was added to cells at 5×10^7^ c.f.u. per well. Plates were then centrifuged at 480 ***g*** for 5 min to initiate contact between *H. pylori* and AGS cells, and incubated for 3 h at 37 °C. After incubation, cell monolayers were washed three times with cold 1× PBS (pH 7.0) to remove non-adherent bacteria, and 0.5 ml F12/10 % FBS/1 % saponin was added to lyse cells. Tenfold serial dilutions of these lysates were plated on blood agar plates and incubated at 37 °C/5 % CO_2_ for 4 days, and titres of the original and post-infection cultures were determined. Relative differences in adherence between strains in multiple independent experiments were evaluated by using Student's *t-*test.

## RESULTS AND DISCUSSION

### Multiple alleles of *sabA* exist in *H. pylori* 26695 and J99 populations due to poly-CT and poly-T tract length variation

**Fig. 1. f1:**
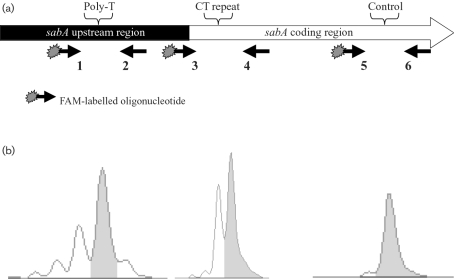
The *H. pylori* strain 26695 population *in vitro* possesses various *sabA* alleles differing in repetitive sequence lengths. (a) Schematic map of the *sabA* locus of *H. pylori* 26695 showing the locations of the promoter region poly-T and coding region CT-repeat tracts, as well as a downstream control region lacking any such putatively hypermutable sequences. The relative locations of oligonucleotide pairs used for AFLP analyses are denoted with black arrows. As indicated, one primer of each pair was 5′ labelled with FAM. (b) Fragment length polymorphism traces indicate five size variants in amplicons of the poly-T region, and four size variants in the amplicons of the CT-repeat tract. The predominant polymorphic form is shaded in grey. A single amplicon length was detected in the downstream control region. This region possesses no repetitive sequences.

The first annotated *H. pylori* genome (strain 26695) identified the *HP0725* locus (subsequently named *sabA*) as containing putatively phase-variable repetitive nucleotide sequences (Tomb *et al.*, 1997). Depending on the length of a CT-repeat tract near the 5′ end of the *sabA* ORF, subsequent translation either encounters a premature termination codon (resulting in a truncated, non-functional gene product), or encodes a full-length functional SabA adhesin protein. Strain 26695 is predicted to contain a 14-base poly-T tract in the promoter region, and to encode an out-of-frame *sabA* allele containing six CT repeats (see schematic diagram, Fig. 1a[Fig f1]). A recent study showed that 49 % of strains examined (*n*=89) were predicted to be in-frame based on the number of CT repeats (de Jonge *et al.*, 2004). However, to our knowledge, no studies have evaluated the degree of diversity at the *sabA* repetitive tracts within a single strain of *H. pylori*, or among multiple strains isolated from a single patient.

We first tested the hypothesis that phase variation could result in *H. pylori* populations containing multiple alleles at the *sabA* poly-T and CT-repeat tracts. Preliminary evidence that the length of the *sabA* CT-repeat region varies in strain 26695 was obtained by electrophoresis of PCR products of the repeat-containing region of *sabA* on a denaturing polyacrylamide gel. When the region was amplified from a plasmid template (pSabA), a single amplicon was observed, while the same amplification using *H. pylori* strain 26695 gDNA as template revealed the presence of a second visible band (data not shown). Next, to better demonstrate allelic variation in the poly-T and CT-repeat tracts of the *sabA* locus, we conducted AFLP analysis. Results of these analyses indicated the presence of alleles of multiple lengths in amplicons containing the *sabA* poly-T and CT-repeat tracts from strain 26695 (Fig. 1). Control amplicons consisting of a portion of the *sabA* locus lacking a repetitive sequence produced a single detectable allele. Analysis of the putatively variable regions amplified from a cloned fragment of *sabA*, pSabA, also revealed a single fragment length (data not shown), thus indicating that the variation seen in AFLP analysis was not an artefact of PCR.

**Fig. 2. f2:**
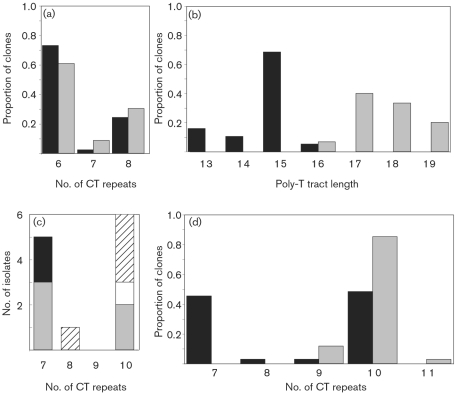
Allelic diversity in poly-T and CT-repeat tracts associated with the *sabA* locus. The poly-T tract is located in the presumed *sabA* promoter region, while the CT-repeat tract lies in the 5′ region of the *sabA* coding sequence. In-frame *sabA* alleles correspond to a dinucleotide tract length of 7 or 10 CT repeats. (a) Distribution of CT-repeat tract lengths in clones amplified from *H. pylori* strain 26695 gDNA (black bars, *n*=41) and cDNA (grey bars, *n*=23). The published strain 26695 genome sequence predicts a tract of six CT repeats (Tomb *et al.*, 1997). (b) Distribution of poly-T tract lengths in PCR amplicons cloned from *H. pylori* 26695 (black bars, *n*=19) and J99 (grey bars, *n*=15). (c) Distribution of CT-repeat tract lengths in J99 re-isolate strains from the antrum (grey bars, *n*=5), cardia (white bar, *n*=1), corpus (hatched bars, *n*=4) and duodenum (black bars, *n*=2). (d) Distribution of CT-repeat tract lengths in clones amplified from low (black bars, *n*=33) or high *in vitro* passage (>50 passages, grey bars, *n*=34) *H. pylori* J99-C6 (re-isolate from corpus biopsy).

To quantify allelic variation at the CT-repeat tract within the *sabA* coding region of *H. pylori* strain 26695, we sequenced pSabA-gDNA-CT clones (Fig. 2a[Fig f2], black bars). Our sequencing results indicated that while 97.6 % (40/41) of the clones confirmed the out-of-frame *sabA* status, 25 % of these clones contained a tract length of eight CT repeats rather than the predicted length of six repeats. A single clone possessed a *sabA* allele containing seven CT repeats; this CT tract length is predicted to express a functional SabA adhesin. These data support the hypothesis that slipped-strand mispairing could generate allelic variation at the *sabA* locus; subsequent environmental pressures could rapidly select for a subpopulation that does or does not express the SabA adhesin.

We next hypothesized that RNA polymerase could err during transcription instead of, or in addition to, mutations induced by slippage of DNA polymerase during replication. As a result of the coupling of transcription and translation in prokaryotes, such an event could have a significant impact on protein expression. This might allow for the potential synthesis of a full-length mRNA transcript, despite the presence of a gDNA sequence that would predict abortive translation. To investigate this possibility, we cloned and sequenced PCR products amplified from *H. pylori* 26695 cDNA synthesized using a *sabA*-specific oligonucleotide (pSabA-cDNA-CT; Fig. 2a[Fig f2], grey bars). As with those derived from a gDNA template, nearly all pSabA-cDNA-CT clones analysed (*n*=23) harboured out-of-frame *sabA* sequences: 91.3 % contained six or eight CT repeats, while two clones possessed an in-frame CT tract with seven repeats. No statistically significant difference in the distribution of *sabA* CT-repeat tract lengths was observed in clones derived from cDNA versus gDNA templates (2 d.f., *χ*^2^=1.76, *P*=0.415). Thus, it seems unlikely that significant expression of SabA could result from the generation of in-frame mRNA transcripts with different CT-repeat tract lengths than the corresponding chromosomal sequence.

Variation in the *sabA* promoter-region poly-T tract was likewise characterized by sequencing pSabA-T clones containing amplicons derived from *H. pylori* 26695 or J99 gDNA (*n*=19 and *n*=15, respectively; Fig. 2b[Fig f2]). Sequencing analysis of all clones containing *sabA* promoter region PCR amplicons from strain 26695 revealed a distribution of 13–16 thymine nucleotides, with a predominant length of 15 bases (68 % of clones). pSabA-T clones derived from strain J99 contained 16–19 thymine nucleotides; 40 % of these had a repeat tract length of 17 bases. Annotated genomic sequences indicate poly-T tracts of 14 nt in 26695 (Tomb *et al.*, 1997), and 18 nt in J99 (Alm *et al.*, 1999). These results provide strong evidence that the *sabA* promoter poly-T tract undergoes slipped-strand mispairing, resulting in a population of *H. pylori* with numerous alleles. Further studies will be needed to ascertain the potential effects of poly-T length variation on transcriptional control at the *sabA* locus.

### Allelic diversity at the *sabA* locus exists within a single patient

In order to gain a better understanding of the actual *sabA* diversity within a single host, we analysed a collection of 12 low-passage strains re-isolated by gastric biopsy from the J99 source patient 6 years after the initial endoscopy (Israel *et al.*, 2001). These strains were isolated from several regions of the stomach: antrum (*n*=5), cardia (*n*=1), corpus (*n*=4), and foci of gastric metaplasia in the duodenum (*n*=2). The *sabA* CT-repeat region of each isolate was amplified by PCR and directly sequenced without cloning in order to assess the presence or absence of variation in the length of the tract relative to the archival strain J99 (Fig. 2c[Fig f2]). Five isolates had a tract of seven CT repeats (in-frame *sabA* allele), one isolate contained eight CT repeats (out-of-frame), and six isolates possessed 10 CT repeats (in-frame).

While the annotated genome sequence of archival strain J99 predicts an out-of-frame *sabA* locus with nine CT repeats (Alm *et al.*, 1999), 11 of the 12 re-isolates analysed were predicted to be in-frame based on a CT-repeat tract length of 7 or 10 repeats This suggested the possibility that a selective pressure favouring expression of SabA may have developed in the host that was not present when the strain was originally isolated. However, we proceeded to sequence the repetitive region of archival J99, as well as two single-colony isolates derived from that strain, and found 10 CT repeats, corresponding to a phase-on *sabA* allele in each case. Taken together, these sequencing results, along with published observations that J99 (but not 26695) binds the Lewis X antigen (Mahdavi *et al.*, 2002), and adherence data in the current study (see below), suggest that J99 does in fact harbour an in-frame *sabA* locus. It is possible that a portion of the *sabA* variation observed in the re-isolates examined in the current study was present at the time strain J99 was initially isolated from an antral biopsy, but not reflected in the published genome sequence. However, as a recent study would suggest (Kuipers *et al.*, 2000), selective pressures in the 6 years between the isolation of the original and novel J99 strains probably contributed to further genetic diversity at *sabA* and other loci.

One isolate (J99 C-6) was selected for additional study of variation in the CT-repeat tract as described above (Fig. 2d[Fig f2]). Plasmid clones (*n*=33) were generated from the amplicons generated from the *sabA* CT-repeat region of *H. pylori* J99 C-6, and sequenced to determine the initial diversity present at the *sabA* locus at the time of the biopsy (‘low passage’). In addition, the effect of prolonged *in vitro* passage on the *sabA* CT-repeat tract was studied in a similar manner using 34 plasmid clones obtained from PCR amplicons of the same region of *sabA* after numerous *in vitro* passages (‘high passage’, >50 passages). In each case, the vast majority of clones (94 % of low-passage and 85 % of high-passage clones; Fisher's exact test, *P*=0.43) contained in-frame *sabA* alleles with 7 or 10 CT repeats. However, a significant shift towards a longer CT-repeat tract was observed in high-passage clones (*χ*^2^ for trend=19.7, d.f*.*=1, *P*<0.0001). These results indicate that a functional SabA was favoured in the gastric niche from which the strain was isolated.

Taken together, the above results clearly demonstrate that phase variation via slipped-strand mispairing occurs at the *H. pylori sabA* locus, and results in the generation of diversity in the length of promoter region poly-T and coding region CT-repeat tracts within a single strain, and among multiple isolates from a single patient. As a result of variations in this repetitive nucleotide tract, populations of *H. pylori* may or may not express the functional SabA adhesin molecules needed to mediate BabA-independent binding to sialyl-Le^x^ antigens on the host cell surface.

### Sensory histidine kinase *arsS* null mutants express a *sabA*-dependent hyper-adherent phenotype

We next sought to study the effects of ArsRS-mediated transcriptional control of *sabA* on adherence by *H. pylori* strains harbouring in-frame (J99) and out-of-frame (26695) *sabA* loci. Several studies have demonstrated that the ArsRS TCST system, in response to environmental changes in pH, regulates the transcription of *sabA* (Dietz *et al.*, 2002; Forsyth *et al.*, 2002; Pflock *et al.*, 2004). We conducted a series of *in vitro* assays that quantified adherence of *H. pylori* strains to AGS cells to test the hypothesis that deletion of the *arsS* histidine kinase locus would result in derepression of *sabA* transcription and, in strains containing in-frame *sabA* CT-repeat tracts, increased adherence to gastric epithelial cells.

**Fig. 3. f3:**
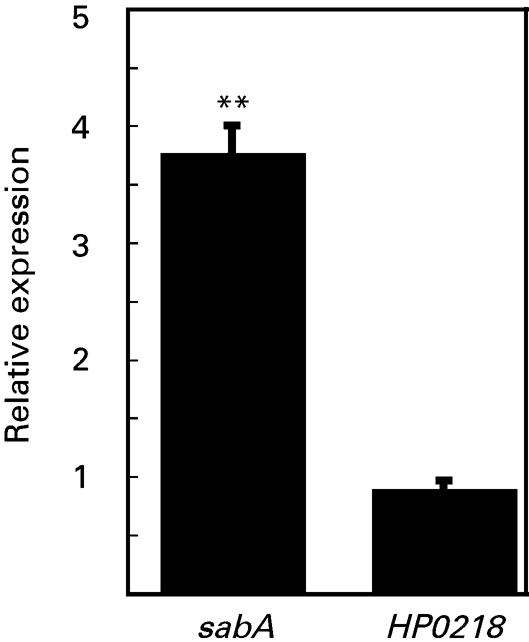
Derepression of *sabA* in J99-*arsS* : : *cat* mutant. Quantitative real-time PCR analysis was performed using cDNA prepared from wild-type *H. pylori* strain J99 and the J99-*arsS* : : *cat* histidine kinase null mutant. Expression levels (mean±sem) of *sabA* and *HP0218* (*arsS*-independent control) relative to J99 wild-type from three independent experiments, each performed in triplicate and normalized to *gyrB*, are presented. Asterisks (**) denote significantly higher *sabA* expression in J99-*arsS* : : *cat* compared to wild-type J99 (*P*=0.0026, Student's *t*-test). No significant difference in *HP0218* expression was detected between the two strains (*P*=0.62, Student's *t*-test).

We first confirmed the transcriptional control of *sabA* by *arsS* (previously demonstrated only by genome-wide transcriptional profiling) by performing quantitative real-time PCR to compare *sabA* transcription in wild-type *H. pylori* and an isogenic *arsS* mutant strain (Fig. 3). Results showed a 3.75±0.25 fold increase in *sabA* cDNA in *H. pylori* J99-*arsS* : : *cat* compared to wild-type J99 (mean±sem from three independent experiments; *P*=0.0026, Student's *t* test). These results are concordant with our earlier DNA macroarray study that indicated that the adhesin *sabA* is 3.34-fold derepressed in the absence of a functional allele of the ArsS histidine kinase (Forsyth *et al.*, 2002). Expression of *HP0218*, a gene not under the transcriptional control of the ArsRS TCST system, was not significantly different in J99 versus J99-*arsS* : : *cat* (*P*=0.62, Student's *t* test).

**Fig. 4. f4:**
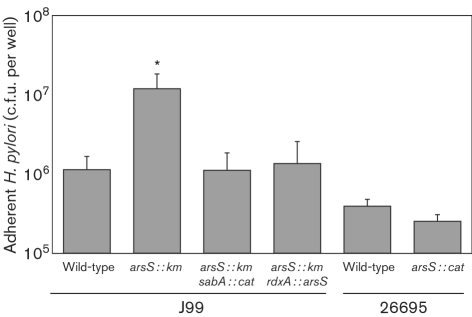
Role of ArsS in *H. pylori* adherence to AGS cells and the function of SabA in augmenting adherence. Disruption of *arsS* (strain J99-*arsS* : : *km*) results in increased adherence of *H. pylori* J99 to AGS cells (* denotes *P*=0.01 vs wild-type, *n*=3 experiments). The increased binding is abrogated by mutation of the *sabA* locus (strain J99-*arsS* : : *km*-*sabA* : : *cat*; *P*=0.97 vs wild-type, *n*=3 experiments) or complementation of the *arsS* locus (strain J99-*arsS* : : *km*-*rdxA* : : *arsS*; *P*=0.75 vs wild-type, *n*=2 experiments). In contrast, disruption of *arsS* in strain 26695, which contains an out-of-frame *sabA* allele, does not result in increased binding. Adherence (c.f.u. per well) data from independent experiments performed in triplicate are presented (mean±sem). Differences between strains in mean adherence were evaluated by using Student's *t*-test.

To examine the functional effect of increased transcription of *sabA* in the absence of *arsS*, we assayed the ability of wild-type *H. pylori* strain J99 and three J99-derived mutant strains (J99-*arsS* : : *km*, J99-*arsS* : : *km-sabA* : : *cat* and J99-*arsS* : : *km*-*rdxA* : : *arsS*) to bind to AGS cells *in vitro* (Fig. 4). Adherence of J99-*arsS* : : *km* to AGS cells was 10.5-fold greater than that of wild-type J99 [1.2(±0.3)×10^7^ vs 1.1(±0.3)×10^6^ c.f.u. per well, *P*=0.01, *n*=3 independent experiments conducted in triplicate]. This increased adherence was reversed when strain J99-*arsS* : : *km* was further modified to either inactivate the *sabA* locus [strain J99-*arsS* : : *km*-*sabA* : : *cat*, 1.1(±0.4)×10^6^ c.f.u. per well; J99-*arsS* : : *km*: 1.2±0.3×10^7^ c.f.u. per well, *n*=3] or reintroduce the *arsS* gene [strain J99-*arsS* : : *km*-*rdxA* : : *arsS*, 1.3(±0.6)×10^6^ c.f.u. per well, J99-*arsS* : : *km*: 1.2±0.3×10^7^ c.f.u. per well,; *n*=2]. No significant difference in mean adherence was detected among J99, J99-*arsS* : : *km*-*sabA* : : *cat* and J99-*arsS* : : *km*-*rdxA* : : *arsS* (*P*>0.75 for all comparisons).

Strain J99-*arsS* : : *km*-*rdxA* : : control, a control strain created by inactivating the *rdxA* gene with an unrelated sequence (Loh & Cover, 2006), retained the hyper-adherent phenotype (data not shown), confirming that the decreased binding seen in strain J99-*arsS* : : *km*-*rdxA* : : *arsS* was due to complementation of *arsS* rather than the inactivation of *rdxA*. A hyper-adherent phenotype similar to that of J99-*arsS* : : *km* was also seen in a J99-*arsS* : : *cat* strain, demonstrating that independently generated *arsS*-null strains possess the same phenotype (data not shown). In contrast, there was no increase in adherence of *H. pylori* strain 26695-*arsS* : : *cat* relative to wild-type 26695 [2.5(±0.3)×10^5^ vs 3.9(±0.5)×10^5^, *P*=0.3, *n*=2]. There was also a trend towards lower basal adherence by wild-type strain 26695 than by J99, potentially due to the lack of SabA-mediated binding in 26695. Further studies will be necessary to elucidate what other factors, such as relative BabA expression, may impact the degree of adherence to AGS cells.

These results establish the functional significance of two distinct mechanisms regulating the expression of the SabA adhesin. The elimination of ArsS in J99 led to a greater than 10-fold increase in adherence that was SabA-dependent, importantly demonstrating that the transcriptional regulation of *sabA* by the ArsRS TCST results in functional changes in *H. pylori* adherence to gastric epithelial cells. However, this elevated binding upon disruption of *arsS* only occurred in strain J99, which contains an in-frame *sabA* allele, but not in strain 26695, which possesses a predominantly out-of-frame *sabA* locus.

Persistent *H. pylori* colonization requires continual adaptation to variations in the gastric microenvironments it inhabits, and to robust host immune and inflammatory responses. Due to the fact that the *H. pylori* genome contains relatively few conserved transcriptional regulators, alternative mechanisms of gene regulation, such as variation in repetitive DNA sequences, play an important role in generating genetic diversity in *H. pylori* (Aras *et al.*, 2003; Mrazek *et al.*, 2007). The present study demonstrates an example of how TCST-mediated regulation and phase variation combine to regulate the transcription of *sabA*, and the subsequent ability of *H. pylori* to bind sialyl-Le^x^ displayed on gastric epithelial cells. The combination of transcriptional regulation of the *sabA* locus by the ArsRS TCST system, and the generation of subpopulations harbouring alternate *sabA* alleles by slipped-strand mispairing during chromosomal replication, could permit *H. pylori* to rapidly adapt to varying microenvironments or host immune responses. The existence of multiple means by which the expression of SabA is controlled in *H. pylori* suggests that precise regulation of this adhesin may be crucial to the virulence of this important pathogen.

## Figures and Tables

**Table 1. t1:** *H. pylori* strains used in this study

**Strain**	**Description**	**Reference**
J99	Clinical isolate, *cagA* positive	Alm *et al.* (1999)
J99-*arsS* : : *km*	*arsS* locus disrupted by kanamycin-resistance cassette	Loh & Cover (2006)
J99-*arsS* : : *cat*	*arsS* locus disrupted by chloramphenicol-resistance cassette	This study
J99-*arsS* : : *km*-*sabA* : : *cat*	J99-*arsS* : : *km* with *sabA* locus disrupted by chloramphenicol-resistance cassette	This study
J99-*arsS* : : *km*-*rdxA* : : *arsS*	J99-*arsS* : : *km* complemented by insertion of *arsS* at the *rdxA* locus, conferring metronidazole resistance	Loh & Cover (2006)
J99-*arsS* : : *km*-*rdxA* : : control	J99-*arsS* : : *km* with control DNA inserted at *rdxA* locus, conferring metronidazole resistance	Loh & Cover (2006)
J99 C-6	Clinical reisolate from corpus biopsy 6 years after isolation of strain J99	Israel *et al.* (2001)
26695	Clinical isolate, *cagA*-positive	Tomb *et al.* (1997)
26695-*arsS* : : *cat*	*arsS* locus disrupted by chloramphenicol-resistance cassette	Forsyth *et al.* (2002)

**Table 2. t2:** Oligonucleotides used in this study

**Name**	**Sequence (5′–3′)**
HP0218forw	GAATTGACCCCTTGAACAATCC
HP0218rev	CGCTAAATTTGGCGGTAACGCTCC
HP0725fwd	GGAACATTTTATGAAAAAGACAATTCTG
HP0725Rev	TGGCTTAATTGCTCGTATTTTTCG
gyrB forw	CGTGGATAACGCTGTAGATGAGAGC
gyrB rev	GGGATTTTTTCCGTGGGGTG
JCN725F	CCAATCAATTTATAGTAAAATTAGGTTCATTG
JCN725R	GGAGGGTTACCCTGGACACTAGCGG
sabAampF	FAM-GGCTCTAGCAATGTGTGGCAGC*
sabArev	AGAATTGTCTTTTTCATAAAATGTTCC
sabAforC	FAM-GAAATCCAATCAATTTATAGTAAAATTAGG*
HP0725R	CGCCGATTTGATAGCCGGCGCTCAC
sabABCFw	ATACCCATTTTGCAACAAGCGGTTACGC
sabABCon	FAM-TAGGCGTTGAACCCGCATTGGG*
HP0725F	CCAATCAATTTATAGTAAAATTAGGTTCATTG
HP0725ForwII	GGAACATTTTATGAAAAAGACAATTCTG
HP0725Ext	GCTTCAACGAAGCCACTTGATT
HP0725Forward	TTTGGCTCTAGCAATGTGTGGC
HP0725Reverse	GGGTTTGATGTTGAGTCTGTTGC

*These oligonucleotides were labelled at the 5′ end with FAM for AFLP analysis. Unlabelled versions of these primers were used for other applications.

## Abbreviations

- AFLP, amplified fragment length polymorphism
- CT repeat, cytosine–thymine repeat
- FAM, 6-carboxyfluorescein
- gDNA, genomic DNA
- poly-T, homopolymeric thymine
- sialyl-Le^x^, sialyl-dimeric Lewis X
- TCST, two-component signal transduction

## References

[r1] Acheson, D. W. & Luccioli, S. (2004). Microbial–gut interactions in health and disease. Mucosal immune responses. Best Pract Res Clin Gastroenterol 18, 387–404. 15123077 10.1016/j.bpg.2003.11.002

[r2] Alm, R. A., Ling, L. S., Moir, D. T., King, B. L., Brown, E. D., Doig, P. C., Smith, D. R., Noonan, B., Guild, B. C. & other authors (1999). Genomic-sequence comparison of two unrelated isolates of the human gastric pathogen *Helicobacter pylori*. Nature 397, 176–180. 9923682 10.1038/16495

[r3] Alper, J. (2001). Searching for medicine's sweet spot. Science 291, 2338–2343. 11269308 10.1126/science.291.5512.2338

[r4] Aras, R. A., Kang, J., Tschumi, A. I., Harasaki, Y. & Blaser, M. J. (2003). Extensive repetitive DNA facilitates prokaryotic genome plasticity. Proc Natl Acad Sci U S A 100, 13579–13584. 14593200 10.1073/pnas.1735481100 PMC263856

[r5] Aspholm, M., Kalia, A., Ruhl, S., Schedin, S., Arnqvist, A., Linden, S., Sjostrom, R., Gerhard, M., Semino-Mora, C. & other authors (2006). *Helicobacter pylori* adhesion to carbohydrates. Methods Enzymol 417, 293–339. 17132512 10.1016/S0076-6879(06)17020-2 PMC2576508

[r6] Beier, D. & Frank, R. (2000). Molecular characterization of two-component systems of *Helicobacter pylori*. J Bacteriol 182, 2068–2076. 10735847 10.1128/jb.182.8.2068-2076.2000 PMC111253

[r7] Bjorkholm, B. & Salama, N. R. (2003). Genomics of *Helicobacter* 2003. Helicobacter 8 (Suppl. 1), 1–7. 10.1046/j.1523-5378.2003.00161.x 14617211

[r8] Blaser, M. J. & Berg, D. E. (2001). *Helicobacter pylori* genetic diversity and risk of human disease. J Clin Invest 107, 767–773. 11285290 10.1172/JCI12672 PMC199587

[r9] Boren, T., Falk, P., Roth, K. A., Larson, G. & Normark, S. (1993). Attachment of *Helicobacter pylori* to human gastric epithelium mediated by blood group antigens. Science 262, 1892–1895. 8018146 10.1126/science.8018146

[r10] Bury-Mone, S., Thiberge, J. M., Contreras, M., Maitournam, A., Labigne, A. & De Reuse, H. (2004). Responsiveness to acidity via metal ion regulators mediates virulence in the gastric pathogen *Helicobacter pylori*. Mol Microbiol 53, 623–638. 15228539 10.1111/j.1365-2958.2004.04137.x

[r11] de Jonge, R., Pot, R. G., Loffeld, R. J., van Vliet, A. H., Kuipers, E. J. & Kusters, J. G. (2004). The functional status of the *Helicobacter pylori sabB* adhesin gene as a putative marker for disease outcome. Helicobacter 9, 158–164. 15068418 10.1111/j.1083-4389.2004.00213.x

[r12] de Vries, N., Duinsbergen, D., Kuipers, E. J., Pot, R. G., Wiesenekker, P., Penn, C. W., van Vliet, A. H., Vandenbroucke-Grauls, C. M. & Kusters, J. G. (2002). Transcriptional phase variation of a type III restriction-modification system in *Helicobacter pylori*. J Bacteriol 184, 6615–6623. 12426350 10.1128/JB.184.23.6615-6623.2002 PMC135423

[r13] Dietz, P., Gerlach, G. & Beier, D. (2002). Identification of target genes regulated by the two-component system HP166–HP165 of *Helicobacter pylori*. J Bacteriol 184, 350–362. 11751811 10.1128/JB.184.2.350-362.2002 PMC139590

[r14] Forsyth, M. H., Cao, P., Garcia, P. P., Hall, J. D. & Cover, T. L. (2002). Genome-wide transcriptional profiling in a histidine kinase mutant of *Helicobacter pylori* identifies members of a regulon. J Bacteriol 184, 4630–4635. 12142435 10.1128/JB.184.16.4630-4635.2002 PMC135264

[r15] Gerhard, M., Lehn, N., Neumayer, N., Boren, T., Rad, R., Schepp, W., Miehlke, S., Classen, M. & Prinz, C. (1999). Clinical relevance of the *Helicobacter pylori* gene for blood-group antigen-binding adhesin. Proc Natl Acad Sci U S A 96, 12778–12783. 10535999 10.1073/pnas.96.22.12778 PMC23096

[r17] Ilver, D., Arnqvist, A., Ogren, J., Frick, I. M., Kersulyte, D., Incecik, E. T., Berg, D. E., Covacci, A., Engstrand, L. & other authors (1998). *Helicobacter pylori* adhesin binding fucosylated histo-blood group antigens revealed by retagging. Science 279, 373–377. 9430586 10.1126/science.279.5349.373

[r18] Israel, D. A., Salama, N., Krishna, U., Rieger, U. M., Atherton, J. C., Falkow, S. & Peek, R. M., Jr (2001). *Helicobacter pylori* genetic diversity within the gastric niche of a single human host. Proc Natl Acad Sci U S A 98, 14625–14630. 11724955 10.1073/pnas.251551698 PMC64732

[r19] Kuipers, E. J., Israel, D. A., Kusters, J. G., Gerrits, M. M., Weel, J., van Der Ende, A., van Der Hulst, R. W., Wirth, H. P., Hook-Nikanne, J. & other authors (2000). Quasispecies development of *Helicobacter pylori* observed in paired isolates obtained years apart from the same host. J Infect Dis 181, 273–282. 10608776 10.1086/315173 PMC2766531

[r20] Kusters, J. G., van Vliet, A. H. & Kuipers, E. J. (2006). Pathogenesis of *Helicobacter pylori* infection. Clin Microbiol Rev 19, 449–490. 16847081 10.1128/CMR.00054-05 PMC1539101

[r21] Lehours, P., Menard, A., Dupouy, S., Bergey, B., Richy, F., Zerbib, F., Ruskone-Fourmestraux, A., Delchier, J. C. & Megraud, F. (2004). Evaluation of the association of nine *Helicobacter pylori* virulence factors with strains involved in low-grade gastric mucosa-associated lymphoid tissue lymphoma. Infect Immun 72, 880–888. 14742532 10.1128/IAI.72.2.880-888.2004 PMC321584

[r22] Linden, S., Boren, T., Dubois, A. & Carlstedt, I. (2004). Rhesus monkey gastric mucins: oligomeric structure, glycoforms and *Helicobacter pylori* binding. Biochem J 379, 765–775. 14736333 10.1042/BJ20031557 PMC1224112

[r23] Livak, K. J. & Schmittgen, T. D. (2001). Analysis of relative gene expression data using real-time quantitative PCR and the 2^−ΔΔ^*^C^*^T^ method. Methods 25, 402–408. 11846609 10.1006/meth.2001.1262

[r24] Loh, J. T. & Cover, T. L. (2006). Requirement of histidine kinases HP0165 and HP1364 for acid resistance in *Helicobacter pylori*. Infect Immun 74, 3052–3059. 16622250 10.1128/IAI.74.5.3052-3059.2006 PMC1459715

[r25] Madrid, J. F., Ballesta, J., Castells, M. T. & Hernandez, F. (1990). Glycoconjugate distribution in the human fundic mucosa revealed by lectin- and glycoprotein-gold cytochemistry. Histochemistry 95, 179–187. 2081692 10.1007/BF00266591

[r26] Mahdavi, J., Sonden, B., Hurtig, M., Olfat, F. O., Forsberg, L., Roche, N., Angstrom, J., Larsson, T., Teneberg, S. & other authors (2002). *Helicobacter pylori* SabA adhesin in persistent infection and chronic inflammation. Science 297, 573–578. 12142529 10.1126/science.1069076 PMC2570540

[r27] Merrell, D. S. & Falkow, S. (2004). Frontal and stealth attack strategies in microbial pathogenesis. Nature 430, 250–256. 15241423 10.1038/nature02760

[r28] Merrell, D. S., Goodrich, M. L., Otto, G., Tompkins, L. S. & Falkow, S. (2003). pH-regulated gene expression of the gastric pathogen *Helicobacter pylori*. Infect Immun 71, 3529–3539. 12761138 10.1128/IAI.71.6.3529-3539.2003 PMC155744

[r29] Mrazek, J., Guo, X. & Shah, A. (2007). Simple sequence repeats in prokaryotic genomes. Proc Natl Acad Sci U S A 104, 8472–8477. 17485665 10.1073/pnas.0702412104 PMC1895974

[r30] Odenbreit, S. (2005). Adherence properties of *Helicobacter pylori*: impact on pathogenesis and adaptation to the host. Int J Med Microbiol 295, 317–324. 16173498 10.1016/j.ijmm.2005.06.003

[r31] Panthel, K., Dietz, P., Haas, R. & Beier, D. (2003). Two-component systems of *Helicobacter pylori* contribute to virulence in a mouse infection model. Infect Immun 71, 5381–5385. 12933888 10.1128/IAI.71.9.5381-5385.2003 PMC187308

[r32] Pflock, M., Dietz, P., Schar, J. & Beier, D. (2004). Genetic evidence for histidine kinase HP165 being an acid sensor of *Helicobacter pylori*. FEMS Microbiol Lett 234, 51–61. 15109719 10.1016/j.femsle.2004.03.023

[r33] Pflock, M., Kennard, S., Delany, I., Scarlato, V. & Beier, D. (2005). Acid-induced activation of the urease promoters is mediated directly by the ArsRS two-component system of *Helicobacter pylori*. Infect Immun 73, 6437–6445. 16177315 10.1128/IAI.73.10.6437-6445.2005 PMC1230922

[r34] Roche, N., Angstrom, J., Hurtig, M., Larsson, T., Boren, T. & Teneberg, S. (2004). *Helicobacter pylori* and complex gangliosides. Infect Immun 72, 1519–1529. 14977958 10.1128/IAI.72.3.1519-1529.2004 PMC356016

[r35] Rubinsztein-Dunlop, S., Guy, B., Lissolo, L. & Fischer, H. (2005). Identification of two new *Helicobacter pylori* surface proteins involved in attachment to epithelial cell lines. J Med Microbiol 54, 427–434. 15824418 10.1099/jmm.0.45921-0

[r36] Sachs, G., Kraut, J. A., Wen, Y., Feng, J. & Scott, D. R. (2006). Urea transport in bacteria: acid acclimation by gastric *Helicobacter* spp. J Membr Biol 212, 71–82. 17264989 10.1007/s00232-006-0867-7

[r37] Salaun, L., Linz, B., Suerbaum, S. & Saunders, N. J. (2004). The diversity within an expanded and redefined repertoire of phase-variable genes in *Helicobacter pylori*. Microbiology 150, 817–830. 15073292 10.1099/mic.0.26993-0

[r38] Saunders, N. J., Peden, J. F., Hood, D. W. & Moxon, E. R. (1998). Simple sequence repeats in the *Helicobacter pylori* genome. Mol Microbiol 27, 1091–1098. 9570395 10.1046/j.1365-2958.1998.00768.x

[r39] Schar, J., Sickmann, A. & Beier, D. (2005). Phosphorylation-independent activity of atypical response regulators of *Helicobacter pylori*. J Bacteriol 187, 3100–3109. 15838037 10.1128/JB.187.9.3100-3109.2005 PMC1082831

[r40] Sheu, B. S., Odenbreit, S., Hung, K. H., Liu, C. P., Sheu, S. M., Yang, H. B. & Wu, J. J. (2006). Interaction between host gastric sialyl-Lewis X and *H. pylori* SabA enhances *H. pylori* density in patients lacking gastric Lewis B antigen. Am J Gastroenterol 101, 36–44. 16405531 10.1111/j.1572-0241.2006.00358.x

[r41] Stock, A. M., Robinson, V. L. & Goudreau, P. N. (2000). Two-component signal transduction. Annu Rev Biochem 69, 183–215. 10966457 10.1146/annurev.biochem.69.1.183

[r42] Tomb, J. F., White, O., Kerlavage, A. R., Clayton, R. A., Sutton, G. G., Fleischmann, R. D., Ketchum, K. A., Klenk, H. P., Gill, S. & other authors (1997). The complete genome sequence of the gastric pathogen *Helicobacter pylori*. Nature 388, 539–547. 9252185 10.1038/41483

[r43] van der Woude, M. W. & Baumler, A. J. (2004). Phase and antigenic variation in bacteria. Clin Microbiol Rev 17, 581–611. 15258095 10.1128/CMR.17.3.581-611.2004 PMC452554

[r44] Yamaoka, Y., Kita, M., Kodama, T., Imamura, S., Ohno, T., Sawai, N., Ishimaru, A., Imanishi, J. & Graham, D. Y. (2002). *Helicobacter pylori* infection in mice: role of outer membrane proteins in colonization and inflammation. Gastroenterology 123, 1992–2004. 12454856 10.1053/gast.2002.37074

[r45] Yamaoka, Y., Ojo, O., Fujimoto, S., Odenbreit, S., Haas, R., Gutierrez, O., El-Zimaity, H. M., Reddy, R., Arnqvist, A. & other authors (2006). *Helicobacter pylori* outer membrane proteins and gastroduodenal disease. Gut 55, 775–781. 16322107 10.1136/gut.2005.083014 PMC1856239

